# Metabolic Heterogeneity in Diffuse Large B-Cell Lymphoma Cells Reveals an Innovative Antimetabolic Combination Strategy

**DOI:** 10.3390/cancers17030394

**Published:** 2025-01-24

**Authors:** Leonardo Lordello, Stéphanie Nuan-Aliman, Karoline Kielbassa-Elkadi, Aurélie Montagne, Konstantina Kotta, Isabelle Martins, Eva Pinto Jurado, Cédric Caradeuc, Jacqueline Lehmann-Che, José Ángel Martínez-Climent, Véronique Meignin, Nicolas Giraud, Guido Kroemer, Gildas Bertho, Catherine Thieblemont, Véronique Baud

**Affiliations:** 1 NF-κB, Differentiation and Cancer, Université Paris Cité, 75006 Paris, France; leomelo@gmail.com (L.L.); stephanie.nuan@gmail.com (S.N.-A.); karoline.elkadi@gmail.com (K.K.-E.); aurelie.montagne@outlook.fr (A.M.); kn.kotta@gmail.com (K.K.); pintoj.eva@gmail.com (E.P.J.); veronique.meignin@aphp.fr (V.M.); catherine.thieblemont@aphp.fr (C.T.); 2 INSERM U1138, Equipe Labellisée Ligue Contre le Cancer, Cordeliers Research Center, Université Paris Cité, Sorbonne Université, 75006 Paris, France; isabellemart@gmail.com (I.M.); kroemer@orange.fr (G.K.); 3 Metabolomics and Cell Biology Platforms, Gustave Roussy Comprehensive Cancer Institute, 94805 Villejuif, France; 4 Laboratory of Pharmacological and Toxicological Chemistry and Biochemistry, LPTCB, Université Paris Cité, Sorbonne Paris Cité, UMR CNRS 8601, 75006 Paris, France; cedric.caradeuc@u-paris.fr (C.C.); nicolas.giraud@u-paris.fr (N.G.); gildas.bertho@u-paris.fr (G.B.); 5 INSERM U976, Immunologie Humaine, Pathophysiologie, Immunothérapie, Université Paris Cité, 75010 Paris, France; jacqueline.lehmann-che@aphp.fr; 6 Service d’Oncologie Moléculaire, AP-HP, Hôpital Saint-Louis, 75010 Paris, France; 7 Department of Hematology, Center for Applied Medical Research, University of Navarra, IDISNA, CIBERONC, 31008 Pamplona, Spain; jamcliment@unav.es; 8 Department of Pathology, AP-HP, Hôpital Saint-Louis, 75010 Paris, France; 9 Pôle de Biologie, Hôpital Européen Georges Pompidou, AP-HP, 75015 Paris, France; 10 Assistance Publique—Hôpitaux de Paris, Hôpital Saint Louis, Hémato-Oncologie, 1 Avenue Claude Vellefaux, 75010 Paris, France

**Keywords:** B-cell lymphoma, diffuse large B-cell lymphoma (DLBCL), combination of antimetabolic drugs, metabolism, oncogenic pathways, cell death, apoptosis

## Abstract

**Background/Objectives:** Diffuse large B-cell lymphoma (DLBCL) is the most common type of non-Hodgkin lymphoma, characterized by aggressive and heterogeneous tumors originating from B-cells. Especially in patients with relapsed or refractory (R/R) disease, DLBCL remains a challenging cancer to treat. Metabolic reprogramming is a hallmark of malignant cells. Our research focuses on developing strategies to enhance clinical outcomes for R/R DLBCL patients by targeting metabolic vulnerabilities. **Methods:** We investigated the effects of combining metformin and L-asparaginase, two FDA-approved antimetabolic drugs, on DLBCL cell metabolism and survival. Nuclear magnetic resonance (NMR) spectroscopy was employed to assess metabolic disturbances induced by the drug combination. The impact on lipid metabolism, glycolysis, glutaminolysis, the tricarboxylic acid (TCA) cycle, and antioxidant responses was examined. Induction of apoptosis was evaluated by FACS analysis. **Results:** The combination of metformin and L-asparaginase strongly sensitized DLBCL cells to apoptosis, independently of their oxidative phosphorylation (OxPhos) or BCR/glycolytic status. NMR spectroscopy revealed that this combination induces broader metabolic disturbances than either drug alone. It disrupts lipid metabolism by altering levels of phospholipids, cholesterol, and fatty acids. Additionally, it counteracts the pro-glycolytic effect of metformin, decreases glycolysis, and reduces glutaminolysis. It also affects the TCA cycle and antioxidant responses, critical for cellular energy production and redox balance. Furthermore, this combination interferes with two key cancer survival pathways, mTORC1 and MAPK signaling. Importantly, proof of principle for its beneficial effect was demonstrated in DLBCL patients. **Conclusions:** Combining metformin and L-asparaginase affects DLBCL cell survival by targeting multiple metabolic pathways and may represent a novel therapeutic approach for R/R DLBCL patients.

## Simple Summary

Diffuse large B-cell lymphoma (DLBCL) is a very common and aggressive form of blood cancer. Despite therapeutic advances, ~40% of DLBCL patients will die. Metabolic reprogramming is a characteristic of cancer cells and a new potential therapeutic target. Our research focuses on developing strategies by targeting metabolic vulnerabilities to improve clinical outcomes for DLBCL patients with therapeutic impasses. Here, we report that combining two antimetabolic drugs, namely metformin and L-asparaginase, induced massive DLBCL cell killing compared to each drug alone. Further, we deciphered how this novel drug combination impacts critical metabolic and oncogenic pathways. Finally, we demonstrated the clinical benefit of this optimized antimetabolic drug treatment for DLBCL patients. Altogether, our data provide a framework for the use of an optimized combination of antimetabolic drugs to overcome resistance in DLBCL patients.

## 1. Introduction

Diffuse large B-cell lymphoma (DLBCL) is the most common lymphoma in adults [1,2]. Even though cure rates have significantly improved since the introduction of anti-CD20 immunochemotherapy, up to 40% of DLBCL patients develop refractory/relapsed (R/R) disease [3]. While cutting-edge immunotherapies by CAR-T cells have improved outcomes, around 60% of R/R DLBCL patients will not be cured by such therapy [4,5]. DLBCL is a highly heterogeneous disease, and the investigation of new therapeutic strategies is urgently needed to guide clinical practice.

Cancer cells exhibit bioenergetic alterations in order to meet their high energy requirements, and deregulation of cellular energetics has been recognized as one of the hallmarks of cancer [6]. Therefore, cancer cell metabolism has opened up new avenues for cancer treatment. High glucose consumption through aerobic glycolysis is referred to as the “Warburg effect”, but it is now realized that deregulated mitochondrial oxidative phosphorylation, amino acid, and lipid metabolism are also critical metabolic hallmarks [7,8,9]. Therefore, the concept of combining molecular-targeted drugs with antimetabolic cooperativity has emerged.

Genetic and functional genomic studies have captured DLBCL heterogeneity and revealed oncogenic drivers in DLBCL [10,11,12,13,14,15,16,17,18]. More recently, metabolic signatures have uncovered distinct metabolic DLBCL subsets, including the OxPhos DLBCL subset characterized by a non-functional BCR and increased mitochondrial metabolism [19], along with the BCR subset that presents an up-regulation of genes encoding BCR signaling components and enzymes associated with glycolysis [19,20]. Due to the emerging complexity of the metabolic landscape of DLBCL, and the high capability of cancer cells to reprogram their metabolism, it can be hypothesized that the inhibition of multiple metabolic pathways using a combination of drugs is necessary for curative potential.

Metformin is a widely prescribed anti-hyperglycemic biguanide drug for type II diabetics, and is also known to inhibit complex I of the mitochondrial electron transport chain [21]. L-asparaginase (Kidrolase^®^) catalyzes the hydrolysis of asparagine and glutamine into aspartate and glutamate, respectively, leading to mitochondrial dysfunction [22,23]. The combination of metformin and L-asparaginase offers a promising therapeutic strategy by simultaneously disrupting mitochondrial oxidative phosphorylation, amino acid metabolism, and glycolysis, thereby exploiting the metabolic heterogeneity and adaptability of DLBCL. However, so far, the impact of such a combination treatment on DLBCL cell survival and metabolic disturbances has never been evaluated.

In the study presented here, we reveal that combining the two FDA-approved antimetabolic drugs, metformin and L-asparaginase, induces significant DLBCL cell apoptosis that was not observed upon treatment with either drug alone, in both OxPhos and BCR/glycolytic DLBCL cells. Furthermore, we uncovered that the combination of metformin and L-asparaginase induces strong metabolic disturbances, including lipid metabolism, glutaminolysis, the TCA cycle, and the redox response. In addition, we demonstrate that the mTOR and MAPK pathways are targeted by this drug combination. Finally, we demonstrated in clinical settings that the combination of metformin with L-asparaginase has a beneficial impact on R/R DLBCL patients. Altogether, our data highlight a previously unrecognized beneficial effect of combining metformin and L-asparaginase to induce DLBCL cell apoptosis, regardless of their cell of origin or metabolic classification. These findings provide a framework for using these two drugs together to overcome resistance in R/R DLBCL patients.

## 2. Materials and Methods

### 2.1. Human DLBCL Cell Lines and Culture Conditions

MD-901, OCI-LY19, and SU-DHL4 DLBCL cell lines were obtained from José Angel Martinez-Climent (Centro de Investigación Médica Aplicada, Pamplona, Spain). Cells were cultured in RPMI-1640 GlutaMAX medium (Gibco, Jenks, OK, USA Life Technologies Limited, Paisley, UK) supplemented with 10% heat-inactivated fetal bovine serum (FBS) (HyClone, Cytiva, Marlborough, MA, USA), 60 µg/mL penicillin, and 100 µg/mL streptomycin (Gibco) at 37 °C with 5% CO_2_.

For glutamine or glucose deprivation experiments, DLBCL cells were grown in RPMI-1640 medium supplemented as above without either glutamine or glucose.

### 2.2. Antibodies and Reagents

Metformin (PHR1084) and 2-deoxy-D-glucose (2-DG, D8375) were purchased from Merck (Darmstadt, Germany), and L-asparaginase (^®^Kidrolase) was a kind gift from Isabelle Madelaine-Chambrin and Nathalie Jourdan (Service de Pharmacie Centrale Hôpital Saint-Louis, Paris, France). The antibodies were purchased from Santa Cruz Biotechnology, Dallas, TX, USA (p70 S6 kinase sc-8418; 4EBP1 sc-9977; p38alpha/beta MAPK sc-7972; ERK1/2 sc-514302; β-actin sc-47798; GAPDH sc-32233), Cell Signaling Technology, Danvers, MA, USA (Phospho-p70 S6 kinase (Thr389) #9234; Phospho-4E-BP1 (Ser65) #9451; Phospho-p38 MAPK (Thr180/Tyr182) #4511; Phospho-p44/42 MAPK (Erk1/2) (Thr202/Tyr204) #4370; 4EBP1 #9452), and Merck (β-actin, A5441).

### 2.3. Measurement of Intracellular ATP Content

Intracellular ATP content was measured using an ATP luminescence detection kit (CellTiter-Glo^®^ Luminescent Cell Viability Assay, Promega, Madison, WI, USA) according to the manufacturer’s instructions. Briefly, 4 × 10^5^ cells were seeded in 100 µL of complete medium. Cells were treated for 1 h with or without 20 mM 2-deoxy-D-glucose (2-DG) at 37 °C followed by 30 min at room temperature to inhibit glycolysis. At the end of the incubation time, 100 µL of CellTiter-Glo solution was added, and cells were incubated for 10 min at room temperature. The luminescence signal (relative luminescence units (RLU)) was recorded with a microplate reader (Centro LB 960 microplate luminometer, Berthold Technologies, Bad Wildbach, Germany). The difference between total ATP content and ATP produced under 2-DG treatment results in glycolytic ATP.

### 2.4. Apoptosis Assays

DLBCL cells were harvested and washed twice with cold PBS. Cells were resuspended in 1X binding buffer containing Annexin V/PE (BD Biosciences Pharmingen, San Diego, CA, USA) and 4′,6-diamidino-2-phenylindole (DAPI, Molecular Probes, Life Technologies, Carlsbad, CA, USA) for 15 min at room temperature. The samples were then subjected to cytometric analysis with a MACSQuant Analyzer 10 Flow Cytometer (Miltenyi Biotec, Bergisch Gladbach, Germany), and the data were analyzed using Flowjo v10.2 software (Ashland, OR, USA).

### 2.5. Measurement of Mitochondrial Transmembrane Potential

For the determination of mitochondrial transmembrane potential (ΔΨm), cells were stained with 100 nM of tetramethylrhodamine, methyl ester (TMRM) (Molecular Probes, Life Technologies, Carlsbad, CA, USA), in RPMI-1640 GlutaMAX medium for 30 min at room temperature. Cells were evaluated by cytometric analysis as above.

### 2.6. Cell Viability Assay

Cell viability was assessed using trypan blue dye exclusion.

### 2.7. Immunoblotting

Whole cell extracts were prepared as previously reported [24]. Proteins (20 μg) were resolved on 7.5% SDS-PAGE-polyacrylamide gels and transferred to nitrocellulose membranes (Cytiva, Marlborough, MA, USA). After blocking with 5% BSA (Merck-Sigma, Louis, MO, USA) in TBST (1X TBS and 0.1% Tween 20 in H_2_O) for 1 h, the membranes were incubated overnight at 4 °C with specific primary antibodies (see Section 2.2). After washing, membranes were incubated with goat anti-mouse or goat anti-rabbit HRP-conjugated secondary antibodies for 1 h at room temperature. Protein bands were visualized using an ECL Western blotting detection kit (Cytiva) and images were captured using an ImageQuant™ chemiluminescence imaging system (Cytiva).

### 2.8. NMR-Based Metabolomics

Exponentially growing DLBCL cells were seeded at a density of 2 × 10^5^–2.5 × 10^5^/mL in a total volume of 25 mL in 75 cm^2^ flasks at the time of treatment with antimetabolic drugs (total 5 × 10^6^ cells/condition) and harvested 24 h later. Decaplicates of either extracellular medium or dry cell pellet samples were prepared for each condition: Extracellular media were added to the deuterated buffer with a 3.57 mM trimethylsilylpropanoic acid (TSP) concentration to obtain a final concentration of 1 mM of the TSP sample in the NMR tube, following standard protocols [25]. Dry cell pellets were treated with 400 µL of ice-cold methanol and 65 µL of ice-cold water. A volume of 200 µL of ice-cold chloroform, then 200 µL of ice-cold water, and finally 200 µL of ice-cold chloroform were further added. The samples were then left on ice for 15 min and centrifugated at 2000 rpm for 15 min. The upper methanol/water phase (containing polar metabolites) and the lower chloroform phase (containing lipophilic compounds) were transferred into separate glass vials. Solvents were removed under a stream of nitrogen for 30 min. The water phases were put in the freeze dryer one night. The dry water phase samples were resuspended in 580 µL of NMR buffer (100 mM sodium phosphate buffer pH 7.4, in D_2_O, with 3.57 mM TSP and 6 mM NaN_3_) to obtain the polar phase extract. The lipophilic phases were resuspended in 600 µL of deuterated NMR solvent (2:1 mixture of pure CDCl_3_ and CDCl_3_ containing 0.03% of TMS, to obtain a final solution of 0.01% of TMS) and then vortexed. After centrifugation (5 min), 580 µL of the supernatant was directly transferred into an NMR tube to prepare the organic phase extract.

All samples were measured on a 500 MHz Bruker Avance III spectrometer equipped with a SampleXpress automation sample changer and a cryogenic 5 mm ^13^C/^1^H dual probe with Z-gradient. The exometabolome sample spectra were acquired using a classical 1D ^1^H experiment used for the metabolomics analysis of biofluids by NMR (1D-NOESY pulse sequence with presaturation for water suppression). The endometabolome samples were acquired using the classical experiment used for the metabolomic analysis of tissue extracts by NMR (1D-CPMG pulse sequence with presaturation for water suppression) [25]. The operating software was TopSpin 3.1 1 (Bruker BioSpin, Ettlingen, BW, Germany).

Metabolite Identification: For each NMR spectrum, the standard processing protocol was applied, including Fourier transform, phase correction, baseline correction, and calibration. Spectra were aligned to a reference signal using TSP as a chemical shift standard (0.016 ppm). For metabolite identification, ^1^H NMR spectra were analyzed using Chenomx NMR Suite 8.0 (Chenomx Inc., Edmonton, AB, Canada). Metabolite identification was performed using the spectral reference library and fitting the peak intensity and location to the observed signals.

Statistical Analysis: The processing of raw data was carried out using NMRProcflow, including baseline correction, chemical shift calibration, data alignment, and variable-sized bucketing [26]. For all datasets, a threshold corresponding to a signal-to-noise ratio of 3 was applied. For normalization, we chose to calculate for each sample the intensity variation between the spectrum of this sample and the reference spectrum recorded on the growing medium (RPMI-1640 left under the same experimental conditions as the sample). In the second step, we normalized this intensity variation with the integration of the number of cells over the duration of the treatment. Subsequently, we used SIMCA 15 software (Umetrics, Umeå, Sweden) to perform principal component analysis (PCA) or orthogonal projections to latent structure discriminant analysis (OPLSDA) in order to discriminate cancer cell line classes. Pareto scaling was used in both analyses. MetaboAnalyst 6.0 [27] was used to perform *t*-tests and determine *p*-values with FDR correction and to generate box plots (https://www.metaboanalyst.ca/, accessed on 7 October 2020).

### 2.9. Antimetabolic Therapy

Three patients followed at the Hôpital Saint-Louis, with a confirmed diagnosis of R/R DLBCL, were treated after consent with an optimized antimetabolic drug combination, including the sequential administration of L-asparaginase and metformin followed by temsirolimus, based on our previous work and our novel preclinical data included in the present study [28]. This combination was discussed and approved on 26 June 2018 by the scientific committee of the Lymphoma Study Association (LYSA). Treatment consisted of L-asparaginase (K, ^®^Kidrolase, Jazz Pharmaceuticals, 10,000 UI/m^2^) over 60 min on days 1, 3, 5, 7, 9, 11, and 13, metformin (M, Mylan, 1000 mg/day) once a day continuously from day 1 to day 14, and temsirolimus (T, ^®^Torisel, Pfizer, 75 mg/week) administered on day 1 of week 3 and week 4. Response assessment was scheduled at month 1 and month 3 based on Lugano criteria [29].

### 2.10. Tissue Processing and Hematoxylin and Eosin Staining of DLBCL Patient Biopsies

Core biopsies obtained from patients with DLBCL were fixed in buffered formalin for 24 h and then transferred to 70% ethanol for 6 h. Subsequently, tissues were dehydrated using increasing concentrations of ethanol (70%, 95%, 100%) in an automated processor (Logos One, Microm France, Brignais, France), followed by a final incubation in isopropanol. After dehydration, tissues were impregnated with paraffin and embedded using a tissue embedding system (Tissue tek), followed by sectioning on a microtome (Autosection, Sakura, Torrance, CA, USA) with a thickness of 4 µm. Slides were stained with hematoxylin and eosin at the department of Anatomic Pathology at Hospital Saint Louis, Paris, France, following standard protocols.

### 2.11. Statistics

Statistical significance was assessed using an unpaired *t*-test (Prism 8.0, GraphPad Software, San Diego, CA, USA). A *p*-value of <0.05 was considered statistically significant, with the following degrees: * *p* < 0.05; ** *p* < 0.01; *** *p* < 0.001; **** *p* < 0.0001.

## 3. Results

### 3.1. Combining Metformin and L-Asparaginase Strongly Enhances Apoptosis in DLBCL Cell Lines Irrespective of Their OxPhos or BCR/Glycolytic Subtypes

**Figure 1:**
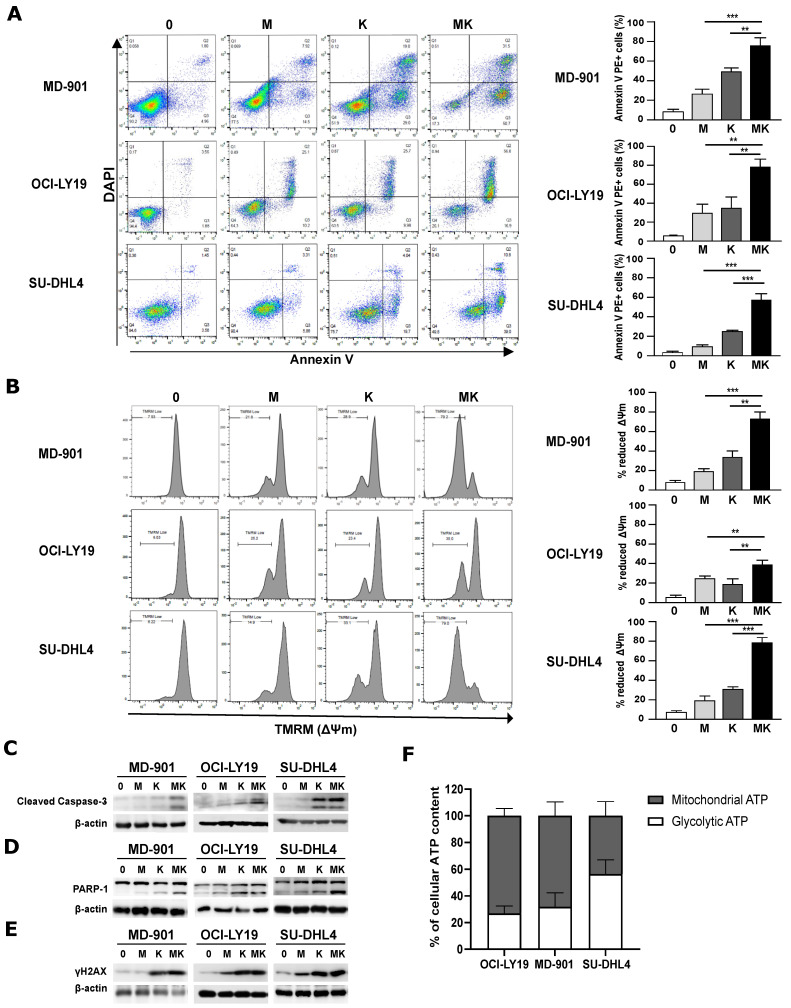
Combining metformin and L-asparaginase strongly enhances apoptosis in DLBCL cell lines irrespective of their OxPhos or BCR/glycolytic subtypes. (**A**) Combination of metformin and L-asparaginase potentiates OxPhos and BCR/glycolytic DLBCL cell apoptosis. MD-901, OCI-LY19, and SU-DHL4 DLBCL cells were treated with the indicated antimetabolic drugs for 48 h and monitored for apoptosis by annexin V-PE and DAPI staining followed by FACS analysis. Error bars are means ± SD (*n* = 3). 0: control; M: metformin (2.5 mM); K: L-asparaginase (2 IU/mL). *p* values by unpaired Student *t*-test, ** *p* < 0.01; *** *p* < 0.001. (**B**) Combination of metformin and L-asparaginase potentiates loss of mitochondrial transmembrane potential (ΔΨm) in DLBCL cells. MD-901, OCI-LY19, and SU-DHL4 DLBCL cells were treated for 36 h with the indicated antimetabolic drugs. DLBCL cells were monitored for loss of mitochondrial transmembrane potential (ΔΨm) by TMRM staining followed by FACS analysis. 0: control; M: metformin (2.5 mM); K: L-asparaginase (2 IU/mL). Error bars represent means ± SD (*n* = 3). *p* values by unpaired Student *t*-test, ** *p* < 0.01; *** *p* < 0.001. (**C**–**E**) Whole cell extracts of MD-901, OCI-LY19, and SU-DHL4 DLBCL cells were treated as in (**A**) for the indicated periods of time and analyzed by immunoblotting for cleaved caspase 3 (**C**), cleaved PARP1 (**D**), and γ-H2AX (**E**). β-actin was also analyzed as loading control (*n* = 3). (**F**) Intracellular levels of glycolytic and mitochondrial ATP of MD-901, OCI-LY19, and SU-DHL4 DLBCL cells were measured as described in Materials and Methods. Error bars represent means ± SD (*n* = 4 individual experiments in duplicates). Original western blots are presented in File S1.

To explore the optimal treatment strategy to target metabolism in DLBCL cells, we evaluated the impact of combining metformin and L-asparaginase, two FDA-approved antimetabolic drugs, on DLBCL cell survival. Using a quantitative measure of apoptosis, we tested each drug individually and in combination. In two GCB models (OCI-LY19 and SU-DHL4) and one ABC model (MD-901), combining metformin and L-asparaginase markedly increased apoptosis compared to each individual drug (Figure 1A). Thus, while individual DLBCL models respond differentially to metformin and L-asparaginase, the combination of the two drugs overcame this functional heterogeneity. This effect was associated with greater loss of mitochondrial transmembrane potential (ΔΨm) (Figure 1B), along with higher levels of caspase 3 and poly(ADP-ribose) polymerase 1 (PARP1) cleavage (Figure 1C,D and Supplementary Materials). Moreover, cells co-treated with metformin and L-asparaginase exhibited a higher level of the DNA-damage marker γH2AX (Figure 1E and Supplementary Materials).

The metabolic status of DLBCL cell lines has been reported to play an important role in drug response [30]. Therefore, we compared the level of glycolytic ATP among the three DLBCL cell lines. The two OxPhos OCI-LY19 and MD-901 DLBCL cell lines presented only a low level of glycolytic ATP (25% and 30%, respectively) compared to the BCR SU-DHL4 DLBCL cell line (~55%) (Figure 1F). This observation is fully in line with their reported sensitivity to the SYK inhibitor R406, which only induces apoptosis in DLBCL cell lines with active BCR signaling, whereas OxPhos DLBCLs that do not display functional BCR signaling are insensitive to this inhibitor [31,32].

Altogether, these data indicate that combining metformin and L-asparaginase induces stronger DNA damage and subsequent apoptosis of DLBCL cells, independently of their GCB, ABC, BCR/glycolytic, and OxPhos subtypes.

### 3.2. Metabolic Alteration Landscape of Metformin and L-Asparaginase Combination in DLBCL Cells

**Figure 2:**
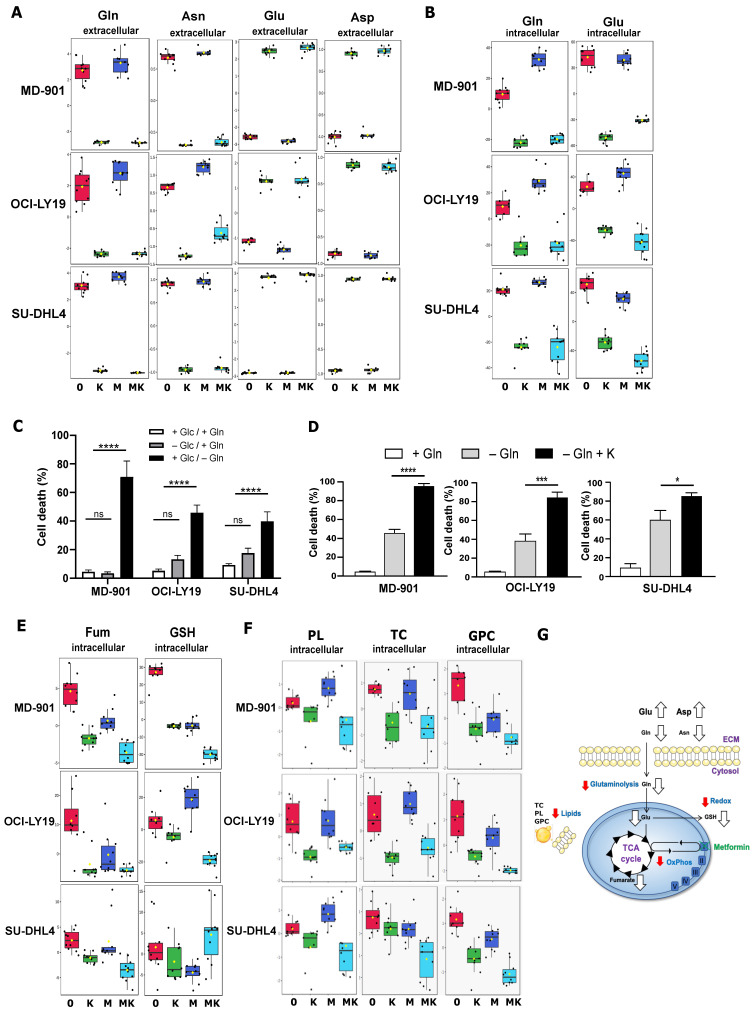
Metabolic alteration landscape of metformin and L-asparaginase combination in DLBCL cells. (**A**,**B**) L-asparaginase exerts dual asparaginase and glutaminase activities in DLBCL cells, strongly impacting glutaminolysis. Metabolic changes in MD-901, OCI-LY19, and SU-DHL4 DLBCL cells were monitored in the extracellular (**A**) and the intracellular (**B**) compartments using an untargeted NMR-based metabolomics approach following treatment for 24 h with metformin (M) (2.5 mM) and L-asparaginase (K) (2 IU/mL) either alone or in combination (MK). Boxplots of relative concentration of the indicated metabolites in the extracellular or intracellular compartments are shown, *n* = 10 per condition. Gln: glutamine; Asn: asparagine; Glu: glutamate; Asp: aspartate. (**C**) Glutamine, but not glucose deprivation, induces DLBCL cell death. MD-901, OCI-LY19, and SU-DHL4 DLBCL cells were cultured in either complete, glutamine-free, or glucose-free medium for 72 h, and cell death was assessed by trypan blue exclusion. Error bars represent means ± SD (*n* = 3). *p* values by unpaired Student *t*-test, **** *p* <0.0001. ns: non-significant. (**D**) L-asparaginase enhances DLBCL cell death under glutamine deprivation. DLBCL cells were cultured for 48 h in either complete medium or glutamine-free medium either in absence or presence of L-asparaginase (2 IU/mL). Cell death was assessed using trypan blue exclusion. Error bars represent means ± SD (*n* = 3). *p* values by unpaired Student *t*-test, * *p* < 0.05; *** *p* < 0.001; **** *p* <0.0001. (**E**) Combination of metformin and L-asparaginase impacts TCA cycle and antioxidant response. Metabolic changes were monitored as in (**A**). Boxplots of relative concentration of indicated metabolites in intracellular compartment are shown, *n* = 10 per condition. Fum: fumarate, GSH: glutathione. (**F**) Combination of metformin and L-asparaginase strongly impacts lipid metabolism. Metabolic changes were monitored as in (**A**). Boxplots of relative concentration of the indicated metabolites in the organic phase of the intracellular compartment are shown, *n* = 10 per condition. PL: phospholipid; TC: total cholesterol; GPC: glycerophosphocholine. (**G**) Schematic representation of the metabolic alteration landscape linked to mitochondrial metabolism upon metformin and L-asparaginase treatment in DLBCL cells.

Using an untargeted NMR-based metabolomics approach, we monitored the metabolic changes occurring in the three DLBCL cell models (two OxPhos and one BCR/glycolytic, see above Figure 1F) following treatment with metformin, L-asparaginase, and their combination for 24 h. Treatment of all three DLBCL cell lines with the *E. coli* L-asparaginase led to a significant depletion not only of asparagine but also of glutamine in the extracellular medium that paralleled the increased levels of aspartate and glutamate, respectively (Figure 2A). These observations indicate that in DLBCL cells, *E. coli* L-asparaginase exerts strong dual asparaginase and glutaminase activities. Most importantly, both glutamine and glutamate were significantly depleted from the intracellular medium, indicating that the *E. coli* L-asparaginase strongly impacts glutaminolysis in DLBCL cells (Figure 2B).

To probe the pro-survival benefit of glutamine in DLBCL cells, we then assessed how glutamine depletion impacts DLBCL cell survival. Remarkably, glutamine deprivation was accompanied by significant DLBCL cell death, in both OxPhos and BCR/glycolytic subtypes (Figure 2C). Notably, treatment of DLBCL cells with the *E. coli* L-asparaginase in the absence of glutamine led to a more pronounced DLBCL cell death compared to glutamine deprivation alone (Figure 2D), suggesting that L-asparaginase treatment targets additional metabolic pathways.

Glutamine is metabolized through glutaminolysis to produce glutamate and α-ketoglutarate, with the latter being used to replenish the tricarboxylic acid (TCA) cycle [7,33]. While limited TCA intermediates could be detected by ^1^H NMR spectroscopy, a significant decrease in fumarate was seen upon L-asparaginase treatment (Figure 2E). Most importantly, co-treatment of L-asparaginase with metformin led to a more pronounced decrease in fumarate compared to treatment with either drug alone (Figure 2E), indicating that this combination more strongly impacts the TCA cycle compared to each drug alone. In line with this, a marked reduction in GSH, a critical antioxidant linked to OxPhos DLBCL cell survival [20], was observed upon co-treatment with metformin and L-asparaginase in the two OxPhos DLBCL cell lines (Figure 2E).

Finally, since lipid metabolism has emerged as critical for cancer cell survival, we also explored the impact of combining metformin and L-asparaginase on lipid levels, analyzing the DLBCL cellular organic phase by ^1^H NMR. A substantial decrease in phospholipids, total cholesterol, and glycerophosphocholine levels was observed only when metformin and L-asparaginase were combined (Figure 2F), again illustrating the broader metabolic impact of combining these two FDA-approved antimetabolic drugs. strongly impacting glutaminolysis, lipid metabolism, the TCA cycle, and redox responses in DLBCL cells (Figure 2G).

Altogether, these observations indicate that combining metformin with L-asparaginase is beneficial for strongly impacting glutaminolysis, lipid metabolism, the TCA cycle, and redox responses in DLBCL cells (Figure 2G).

### 3.3. Combination of Metformin and L-Asparaginase Strongly Inhibits Glycolysis

**Figure 3:**
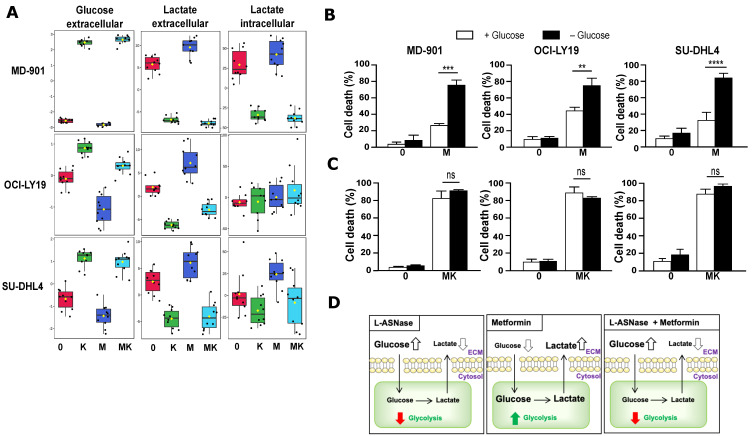
Combination of metformin and L-asparaginase strongly inhibits glycolysis. (**A**) L-asparaginase inhibits glycolysis in DLBCL cells and counteracts the pro-glycolytic effect of metformin. Metabolic changes were monitored using an untargeted NMR-based metabolomics approach. Boxplots of relative concentration of the indicated metabolites in the extracellular or intracellular compartments are shown, *n* = 10 per condition. (**B**) Glucose limits the cytotoxic effect of metformin in DLBCL cells. DLBCL cells were cultured for 48 h in either complete medium (+ Glucose) or glucose-free medium (-Glucose) either in absence or presence of L-asparaginase (2 IU/mL). Cell death was assessed using trypan blue exclusion. Error bars represent means ± SD (*n* = 3). *p* values by unpaired Student *t*-test, ** *p* < 0.01; *** *p* < 0.001, **** *p* <0.0001. (**C**) Combination of metformin and L-asparaginase abolishes glucose-dependent pro-survival effect in DLBCL cells. DLBCL cells were cultured for 48 h in either complete medium or glucose-free medium either in absence or presence of combination of metformin (2.5 mM) and L-asparaginase (2 IU/mL). Cell death was assessed using trypan blue exclusion. Error bars represent means ± SD (*n* = 3). *p* values by unpaired Student *t*-test, ns: not significant. (**D**) Schematic representation of glycolysis alteration upon metformin and L-asparaginase treatment in DLBCL cells.

Both increased glucose uptake and glycolysis, which converts glucose into lactate under aerobic conditions (also known as the “Warburg effect”) have been reported in DLBCL cells [34]. To understand the beneficial effect of combining metformin and L-asparaginase on DLBCL cells, we used ^1^H NMR to evaluate how each drug alone or in combination impacts glycolysis by measuring glucose and lactate levels. Metformin treatment resulted in decreased glucose and increased lactate levels in the extracellular medium, as expected for an anti-diabetic drug (Figure 3A). In contrast, surprisingly, L-asparaginase inhibited glycolysis, as indicated by a significant increase in glucose and a decrease in lactate levels in the extracellular medium (Figure 3A). Importantly, combining L-asparaginase with metformin counteracted the pro-glycolytic effect of metformin treatment alone, highlighting the beneficial antimetabolic effect of the combination of metformin with L-asparaginase (Figure 3A).

Glucose depletion sensitized all three DLBCL cell models (two OxPhos and one glycolytic) to the cytotoxic effect of metformin (Figure 3B), indicating that metformin-induced glycolysis provides a survival signal to DLBCL cells. Most importantly, the combination of L-asparaginase with metformin counteracted the glucose-dependent pro-survival effect of metformin treatment alone (Figure 3B,C).

Collectively, our findings demonstrate that combining metformin with L-asparaginase enhances their pro-apoptotic efficacy in both OxPhos and glycolytic DLBCL by cooperatively targeting multiple metabolic pathways (Figure 2G and Figure 3D).

### 3.4. Metformin Cooperates with L-Asparaginase to Modulate mTOR and MAPK Signaling Pathways

**Figure 4:**
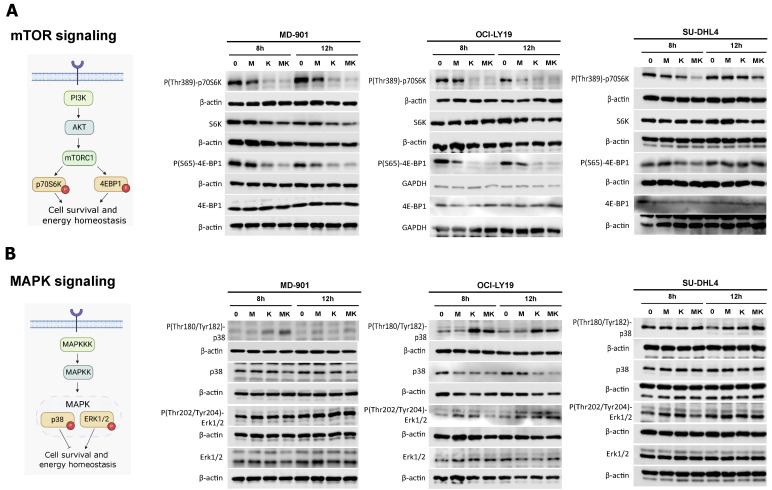
Metformin cooperates with L-asparaginase to modulate mTOR and MAPK signaling pathways. (**A**,**B**) Whole cell extracts of MD-901, OCI-LY19, and SU-DHL4 DLBCL cells were treated with metformin (M) (2.5 mM) and L-asparaginase (K) (2 IU/mL) either alone or in combination (MK) for the indicated periods of time and analyzed for (**A**) mTORC1 activity and (**B**) p38 and ERK MAPK pathways, using the indicated antibodies. Glyceraldehyde-3-phosphate dehydrogenase (GAPDH) and β-actin were also analyzed as loading controls. The pathway diagrams were generated using BioRender (www.biorender.com). Original western blots are presented in File S1.

Since the combination of metformin with L-asparaginase induces massive DLBCL cell apoptosis and disrupts various pro-survival metabolic pathways, including glutamine metabolism, lipid metabolism, and mitochondrial activity, we next investigated how these two antimetabolic drugs impact critical oncogenic pathways involved in the metabolic stress response and cell survival. The mammalian target of rapamycin complex 1 (mTORC1) is a central regulator of energy homeostasis and cell survival [35]. Metformin and L-asparaginase together markedly decreased mTORC1 activity, as evidenced by reduced phosphorylation of p70-S6K on threonine 389 and 4EBP1 on serine 65 (Figure 4A). Interestingly, the combination of metformin and L-asparaginase modulates mTORC1 in the two OxPhos DLBCL cell lines (MD-901 and OCI-LY19) and the glycolytic one (SU-DHL4).

To further elucidate the mechanisms underlying the action of metformin and L-asparaginase, we investigated their impact on p38 and ERK MAP kinases, which are critical regulators of cell survival under stress and key modulators of cellular metabolism [36]. Treatment with L-asparaginase and metformin strongly increased p38 activity, as indicated by increased phosphorylation on threonine 180 and tyrosine 182, while having minimal to no effect on ERK activity (Figure 4B).

### 3.5. Combination of Metformin and L-Asparaginase Has a Clinical Benefit in Refractory/Relapsed DLBCL Patients

**Figure 5:**
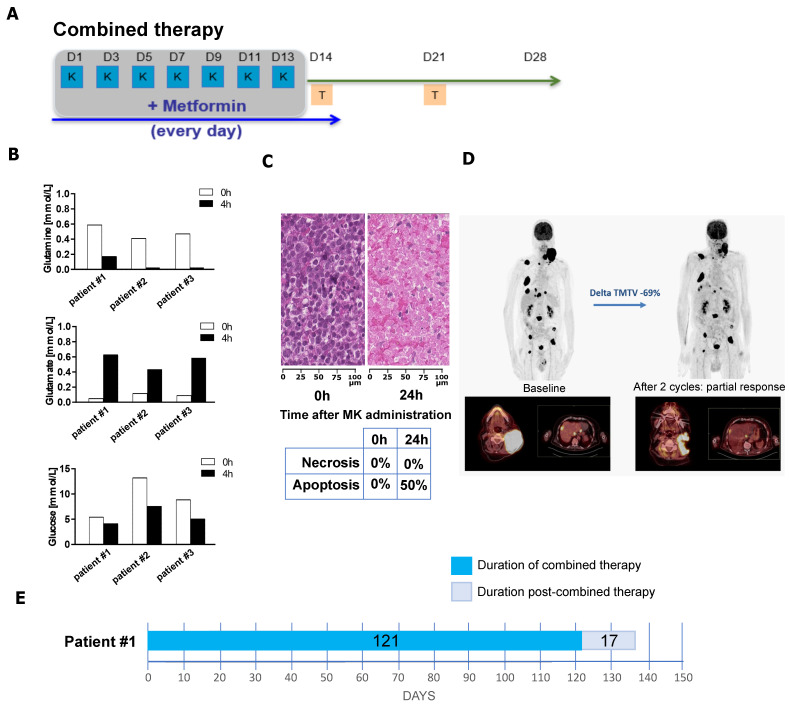
Combination of metformin and L-asparaginase has a clinical benefit in refractory/relapsed DLBCL patients. (**A**) Schematic representation of a four-week cycle of the optimized combined antimetabolic treatment. Metformin (M) and L-asparaginase (K, ^®^Kidrolase) were administered during the first two weeks, and the mTORC1 inhibitor temsirolimus (T, ^®^Torisel) in the subsequent two weeks. Specifically, L-asparaginase (10,000 IU) was administered on days 1, 3, 5, 7, 9, 11, and 13, and metformin (1000 mg/day) daily from day 1 to day 14. Temsirolimus (25 mg) was administered twice on days 14 and 21. (**B**) Reduced levels of glucose and glutamine, along with increased levels of glutamate in the serum of the three analyzed patients treated with the antimetabolic therapy after 4 h of co-administration of metformin and L-asparaginase. (**C**) Induction of apoptosis after 24 h of co-administration of metformin and L-asparaginase in one patient. (**D**) PET/CT analysis of one patient before and after 2 cycles of the antimetabolic treatment showing partial response with a reduction of 69% of total metabolic tumor volume (TMTV). (**E**) Response to treatment for the patient analyzed in (**D**). Duration of treatment under the antimetabolic treatment (dark blue). Duration of total survival after the treatment (light blue). The treatment stopped because of progression.

Since currently available mouse models do not fully recapitulate human DLBCL as they lack key features, such as markers of mature B-cells, it was important to validate the benefit of combining metformin and L-asparaginase in human DLBCL patients. Therefore, we set up a combined antimetabolic therapy protocol as described in Figure 5A. The combination was not administered longer than 14 days to limit L-asparaginase toxicities. During the final two weeks of each antimetabolic treatment cycle, patients received temsirolimus (Torisel), an mTOR inhibitor, to target the pathway affected by the combination of L-asparaginase and metformin (Figure 4A). The three treated patients were refractory to R-CHOP and immunotherapy; one of these patients had also failed CAR-T cell therapy. All three patients exhibited reduced serum levels of glucose and glutamine and increased serum levels of glutamate four hours after the administration of metformin and L-asparaginase (Figure 5B). Notably, one patient showed approximately 50% apoptosis in a core tumor biopsy performed 24 h after metformin and L-asparaginase administration (Figure 5C). This patient experienced a 69% reduction in total metabolic tumor volume (TMTV) as measured by positron emission tomography (PET) after 56 days of treatment (Figure 5D). This patient completed two cycles, which were extended to a total of 121 days (Figure 5E), achieving a response duration of 68 days. Altogether, although the sample size was small, these data established proof of principle that patients refractory to immunochemotherapy or cell immunotherapy, such as CAR-T cells, can benefit from an optimized antimetabolic targeting strategy.

## 4. Discussion

Reprogramming of cancer cell metabolism is now recognized as a hallmark of cancer [6]. Furthermore, cancer cells have a high capacity to adapt their metabolism to their environmental conditions [9]. Thus, an interesting therapeutic strategy in cancer could involve the combination of antimetabolic drugs capable of promoting antimetabolic cooperativity to induce cell death [37]. In the study presented here, we conducted the first comprehensive analysis of how the combination of the two FDA-approved drugs, metformin and L-asparaginase, impacts DLBCL cell metabolism and survival, both in cellulo and in vivo. Remarkably, we found that the combination of metformin with L-asparaginase induces a much broader spectrum of metabolic disturbances than that observed with each drug alone. First, we demonstrated that combining L-asparaginase with metformin strongly impacts lipid metabolism. Second, L-asparaginase is able to nullify the pro-glycolytic effect of metformin, with the combination of the two leading to a massive decrease in glycolysis. Third, L-asparaginase in the presence of metformin optimally reduces glutaminolysis. Fourth, the TCA cycle and antioxidant response are strongly impacted by the combination of L-asparaginase and metformin. Fifth, the combination of L-asparaginase with metformin impacts the mTORC1 and MAPK pathways. The impact of treatment with metformin and L-asparaginase on levels of phospholipids, cholesterol, and fatty acids was somewhat unexpected, even though metformin alone is known to affect lipid metabolism [38]. Nonetheless, it is of great importance since lipid metabolism is one of the most altered metabolic pathways in multiple cancer types [8,39]. Further, recent studies have implicated fatty acid metabolism as a major source of energy in aggressive B-cell lymphoma, including DLBCL [20,40,41,42]. Still, the exact molecular mechanisms underlying these metabolic alterations and their relation to the inferior clinical outcome of patients have remained largely elusive, which is worth further investigation.

We demonstrated that the combination of metformin with L-asparaginase triggers massive DLBCL cell apoptosis and DNA damage compared to what was seen with each antimetabolic drug alone. The beneficial combinatory effect was seen in both OxPhos and BCR/glycolytic DLBCL cell lines, thus highlighting the benefit of inducing a wide spectrum of metabolic disturbances to promote DLBCL cell apoptosis. Importantly, massive induction of apoptosis was also seen in DLBCL patient tumor biopsies after the co-administration of L-asparaginase with metformin. Recently, mechanisms of compensatory energy acquisition pathways in glycolysis-suppressed DLBCL cells have been explored. Silencing of Glyceraldehyde-3-phosphate dehydrogenase (GAPDH), a critical enzyme regulating glycolysis, in Eμ-Myc-GAPDH^high^ cells isolated from mouse primary B lymphomas (used as a model of aggressive non-Hodgkin B-cell lymphomas) led to a metabolic switch towards glutamine and OxPhos metabolism. Conversely, overexpression of GAPDH in Eμ-Myc-GAPDH^low^ cells was sufficient to induce a metabolic switch towards glycolysis [28]. Moreover, the GAPDH-dependent metabolic reprogramming strongly limited the capacity of either phenformin or L-asparaginase to induce apoptosis of Eμ-Myc murine lymphoma cells [28]. Therefore, these observations are fully consistent with our study, which reveals that targeting various metabolic pathways is required for optimal induction of DLBCL cell death in OxPhos and BCR/glycolytic human DLBCL cells.

Altogether, our data point to metformin and L-asparaginase as a promising combinatory strategy to induce massive DLBCL cell apoptosis. Our study reinforces the concept that combining antimetabolic drugs will be necessary to overcome cancer cell resistance through energy metabolism reprogramming and to ensure a long-term anticancer response.

Many clinical trials using individual metabolic inhibitors have failed to improve the outcome of DLBCL patients [43,44,45,46,47,48,49,50]. This is likely not due to poor efficacy of the drugs, but rather to the ability of cancer cells to find a metabolic path to reprogram and bypass the targeted metabolic pathway. The use of metformin led to inconclusive results regarding its beneficial impact on DLBCL patient outcome [47,48,49,50]. L-asparaginase alone was not evaluated in DLBCL patients. A previous study revealed a beneficial impact of administering L-asparaginase combined with an mTOR inhibitor, temsirolimus, followed by the administration of metformin in four R/R DLBCL patients exhibiting an OxPhos profile (GAPDH^low^) [28]. In our combined antimetabolic therapy, the sequence of administration was optimized based on our preclinical study with L-asparaginase being co-administered with metformin, followed by the administration of the mTOR inhibitor. Remarkably, one of the patients presented a 69% reduction in TMTV after two cycles of treatment, even though this patient was R/R to nine prior lines of treatment, including CAR-T cells. Our data indicate that administering an optimized combination of antimetabolic drugs based on a deeper understanding of DLBCL cell metabolism may open new avenues for R/R DLBCL patients.

## 5. Conclusions

In summary, we established that combining metformin with L-asparaginase induces a large spectrum of metabolic disturbances, including lipid, glutamine, and mitochondrial metabolism as well as glycolysis. Further, it induces massive DLBCL cell apoptosis both in vitro and in vivo in DLBCL patients. Our data are of great functional importance because they constitute a significant advance in understanding the metabolic vulnerabilities of DLBCL cells. They provide a strong rationale for combinatory therapeutic interventions that include optimized combinations of antimetabolic drugs in R/R DLBCL patients.

## Data Availability

Data are all contained within the article.

## Supplementary Materials

The following supporting information can be downloaded at https://www.mdpi.com/article/10.3390/cancers17030394/s1. File S1: Original western blots.
