# Supplementary material for: Metabolic Heterogeneity in Diffuse Large B-Cell Lymphoma Cells Reveals an Innovative Antimetabolic Combination Strategy
Source: Cancers (Basel). 2025 Jan 24;17(3):394. doi: 10.3390/cancers17030394 (PMC11816127; doi:10.3390/cancers17030394)
Supplement: Supplementary file 1 [file cancers-17-00394-s001.zip › cancers-3357279-supplementary.pdf]

Figure 1C

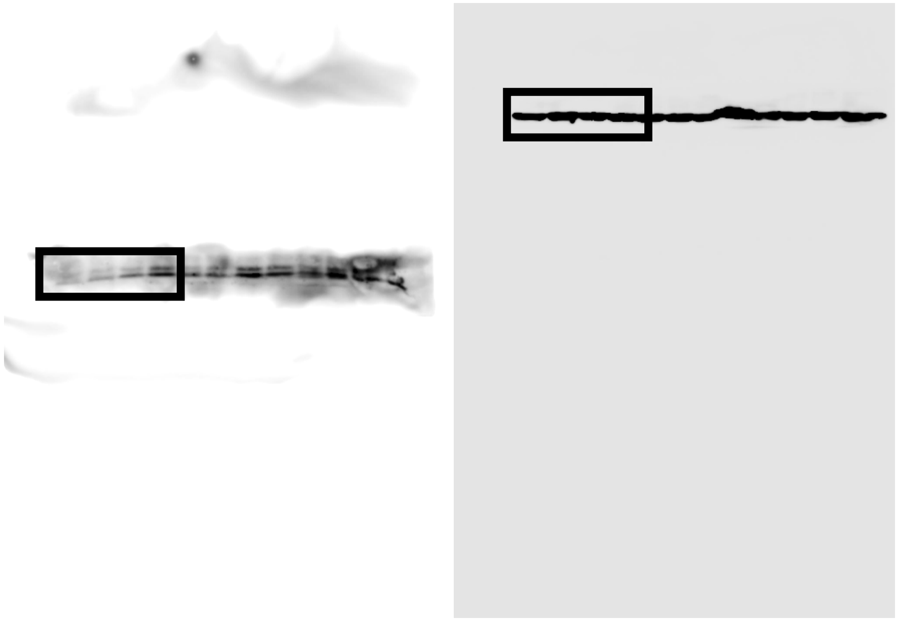

Lordello, L. et al.

Figure 1C

MD-901

IB: Cleaved Caspase-3

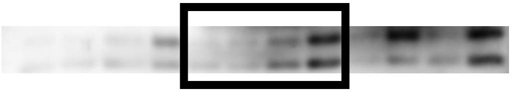

IB: β-actin

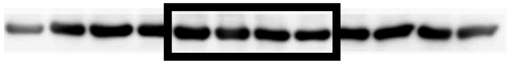

Lordello, L. et al.

Figure 1C

SU-DHL4

IB: Cleaved Caspase-3

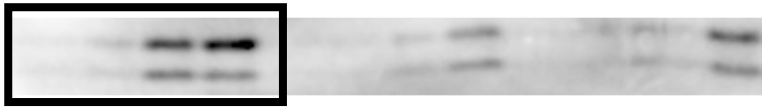

IB: β-actin

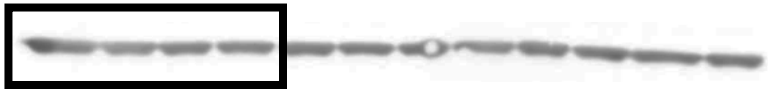

Lordello, L. et al.

Figure 1D

OCI-LY19

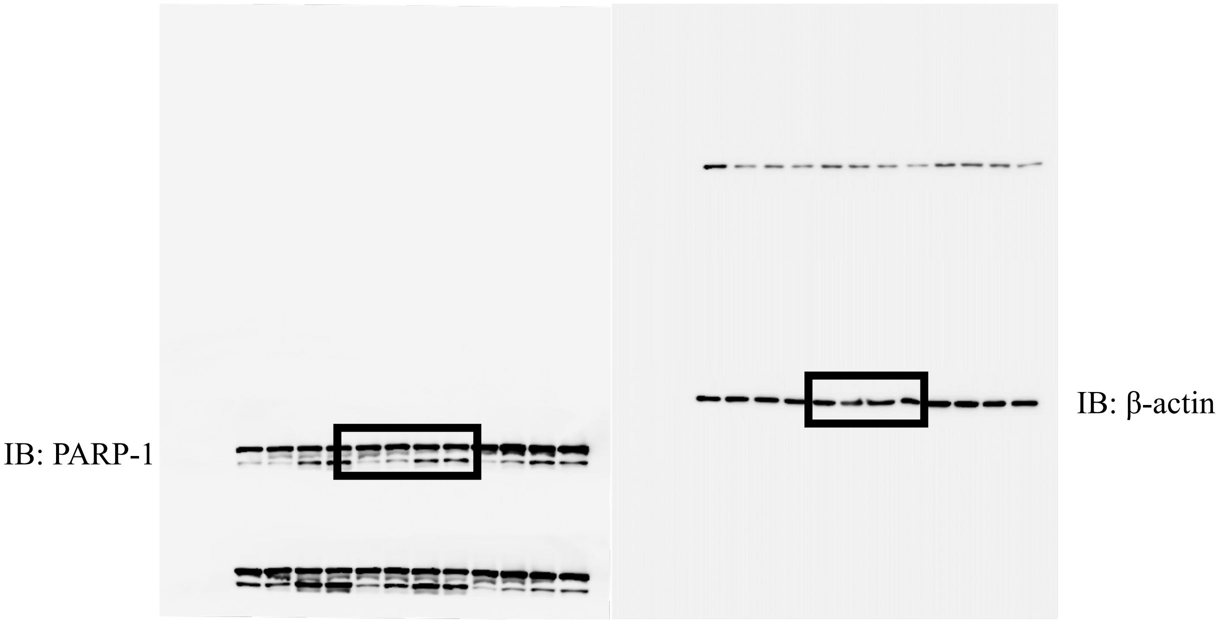

Lordello, L. et al.

Figure 1D

MD-901

IB: β-actin

IB: PARP-1

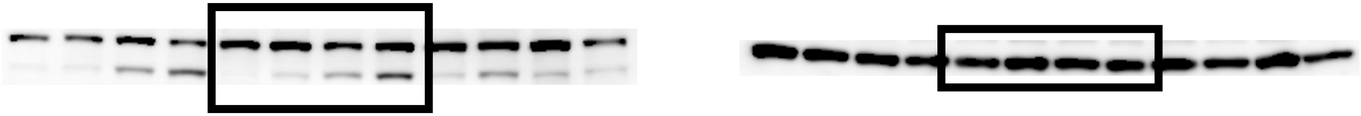

Lordello, L. et al.

Figure 1D

SU-DHL4

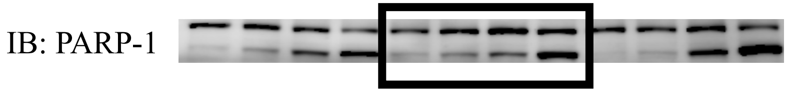

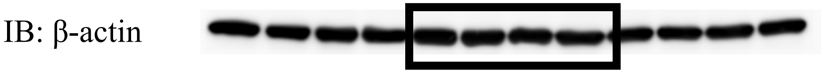

Lordello, L. et al.

Figure 1E

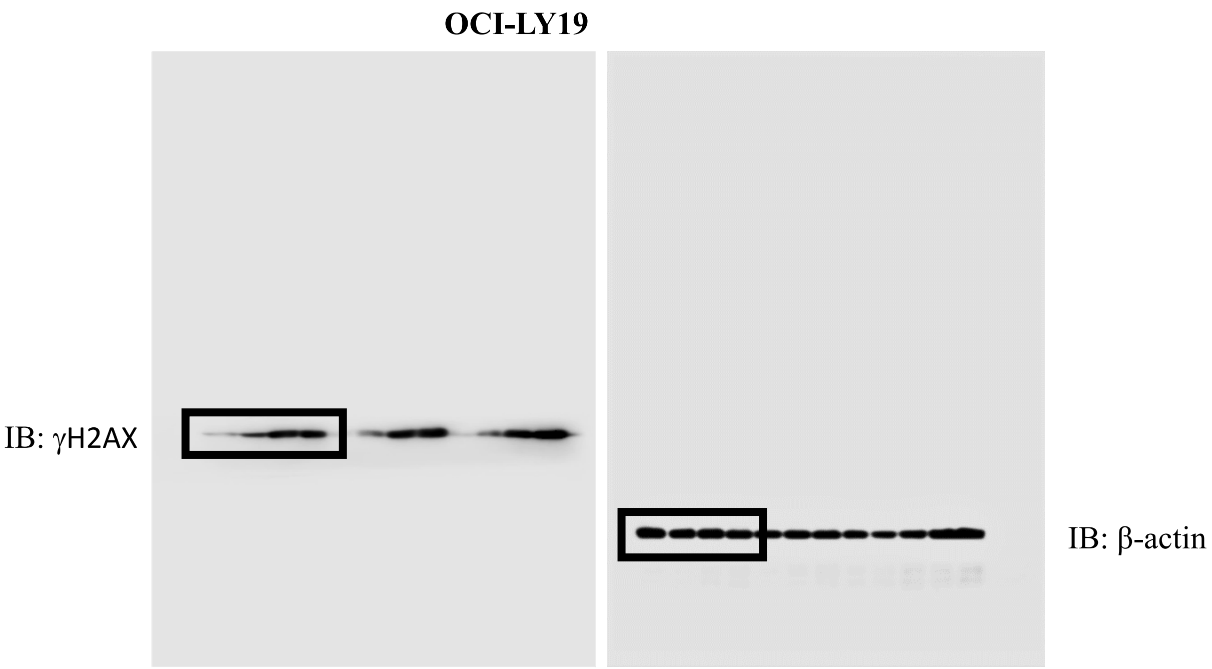

Lordello, L. et al.

Figure 1E

MD-901

IB: γH2AX

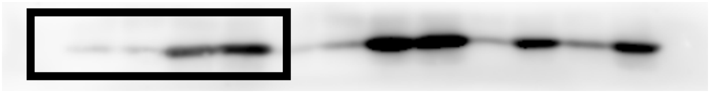

IB: β-actin

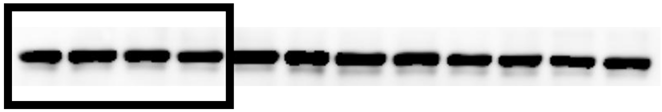

Lordello, L. et al.

Figure 1E

SU-DHL4

IB: β-actin

IB: γH2AX

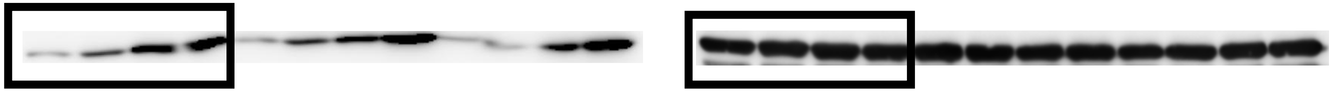

Lordello, L. et al.

Figure 4A

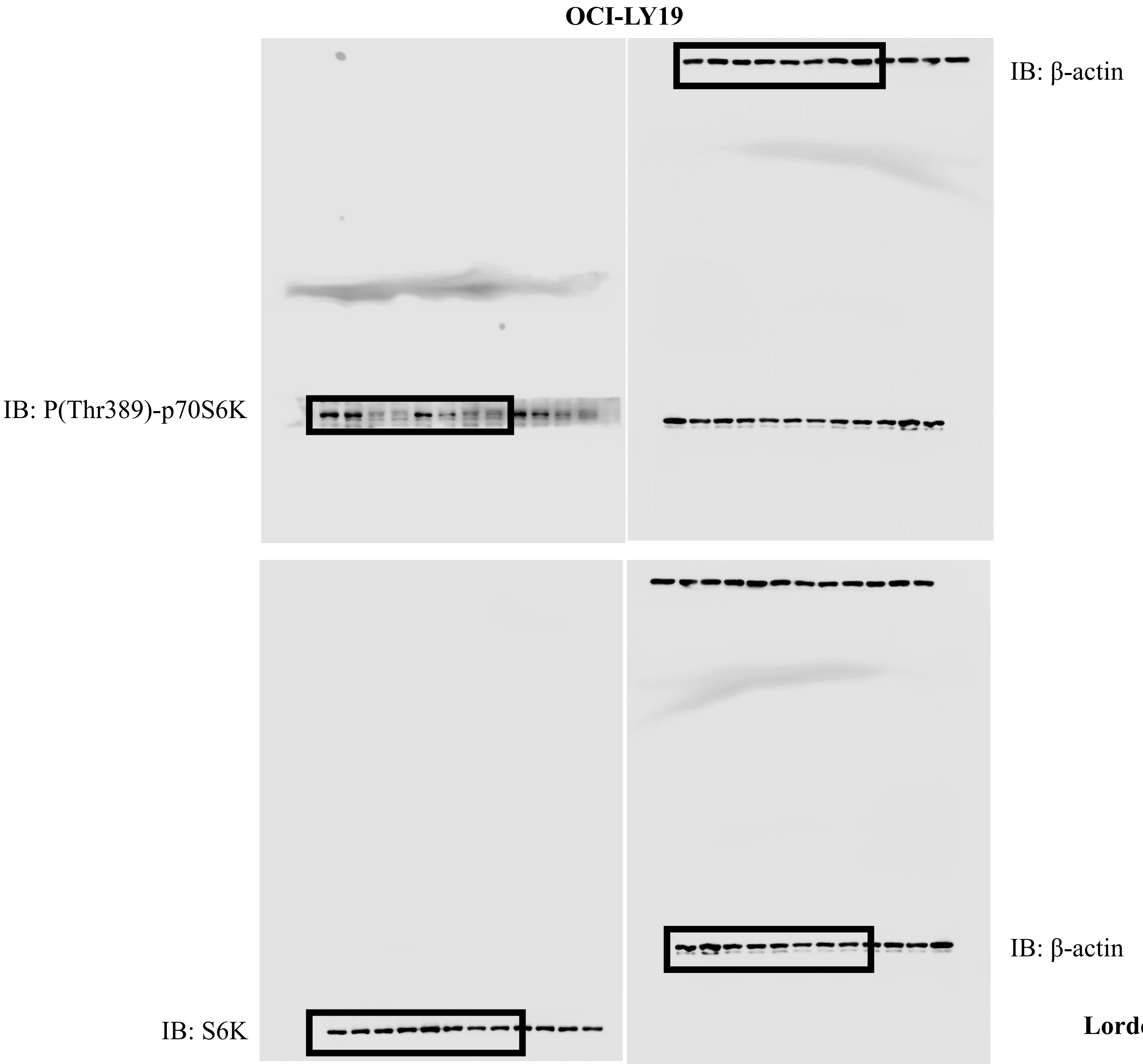

Lordello, L. et al.

Figure 4A

OCI-LY19

IB: P(S65)-4E-BP1

IB: 4E-BP1

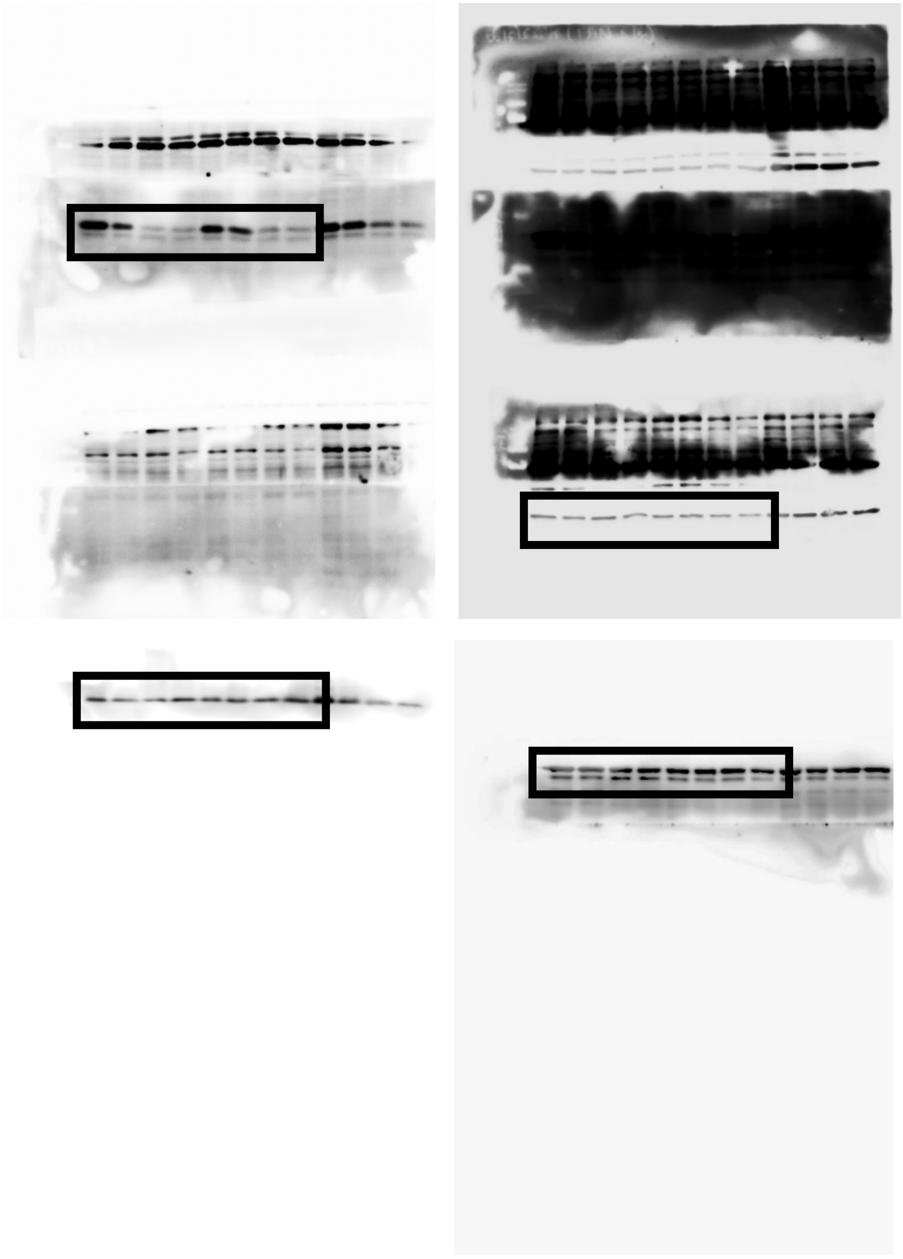

IB: GAPDH

IB: GAPDH

Lordello, L. et al.

Figure 4A

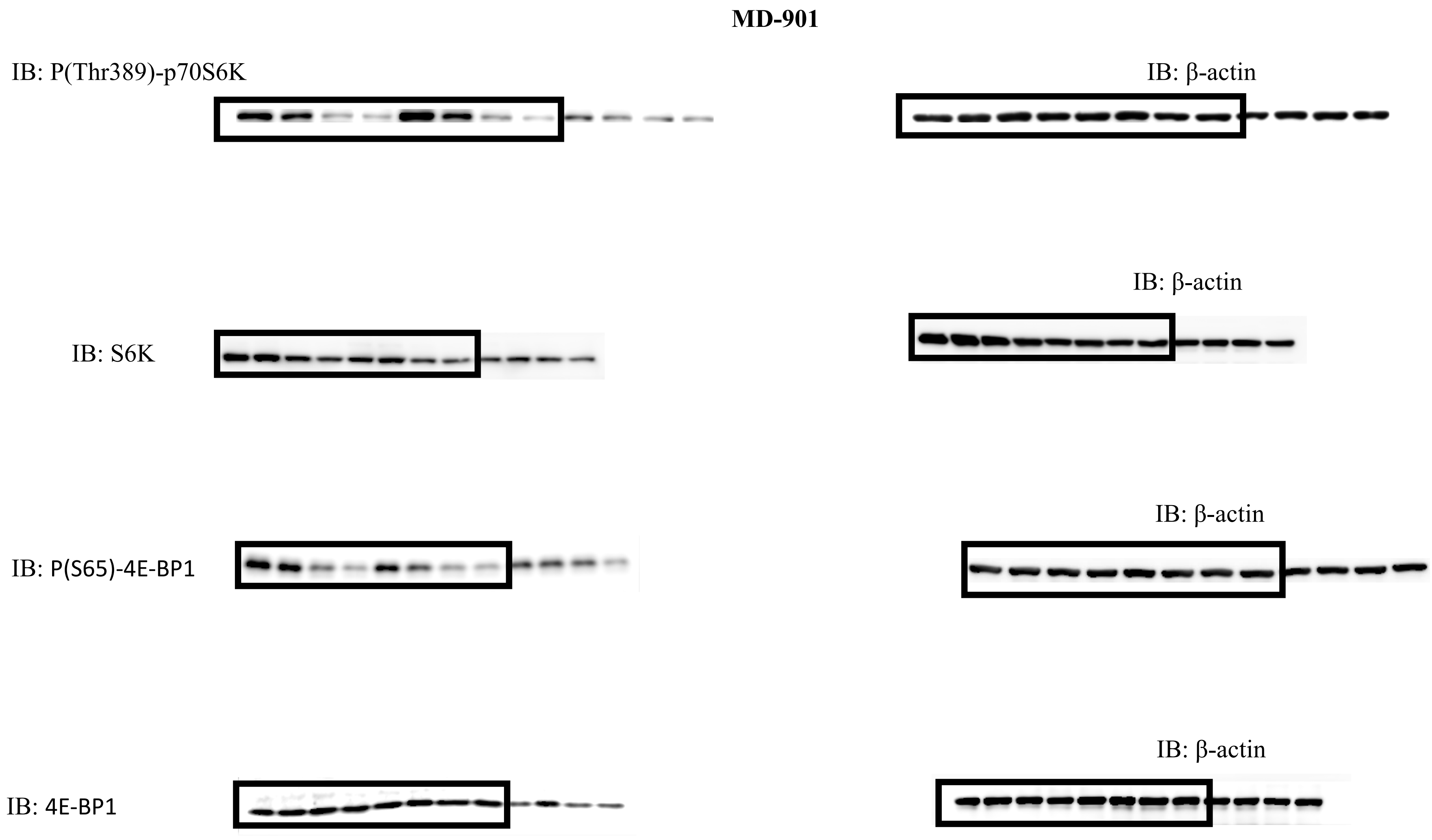

Lordello, L. et al.

Figure 4A

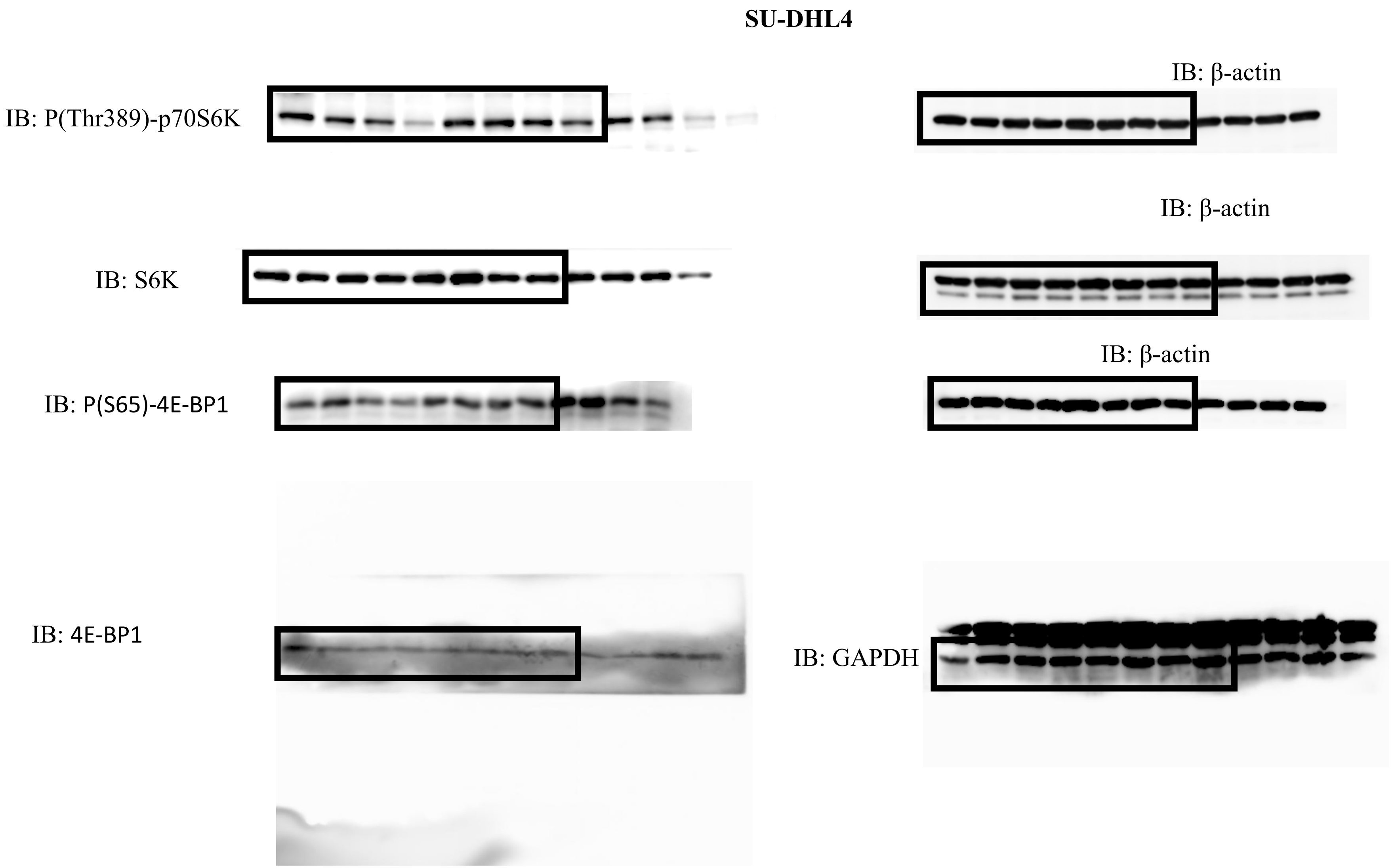

Lordello, L. et al.

Figure 4B

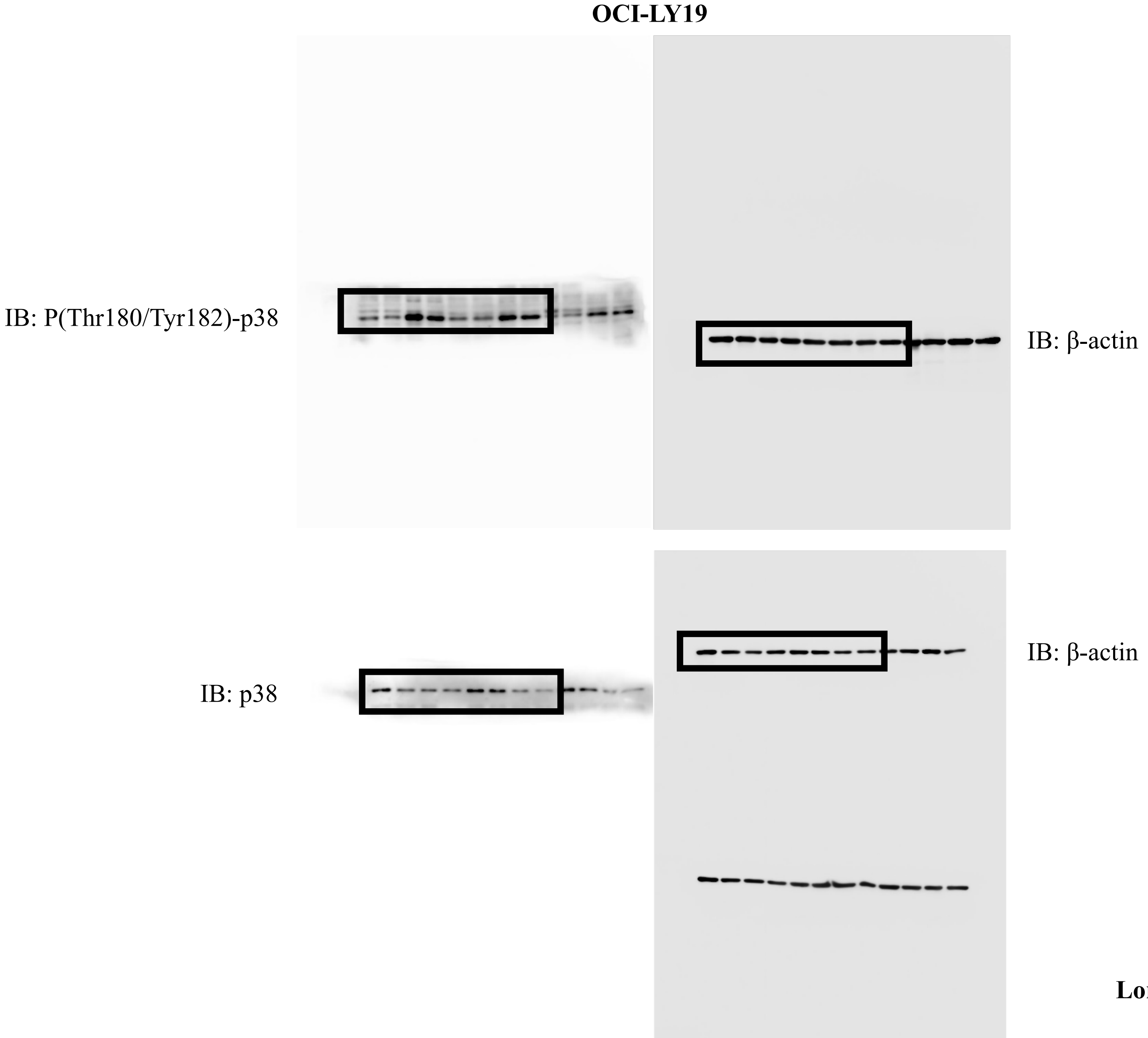

Lordello, L. et al.

Figure 4B

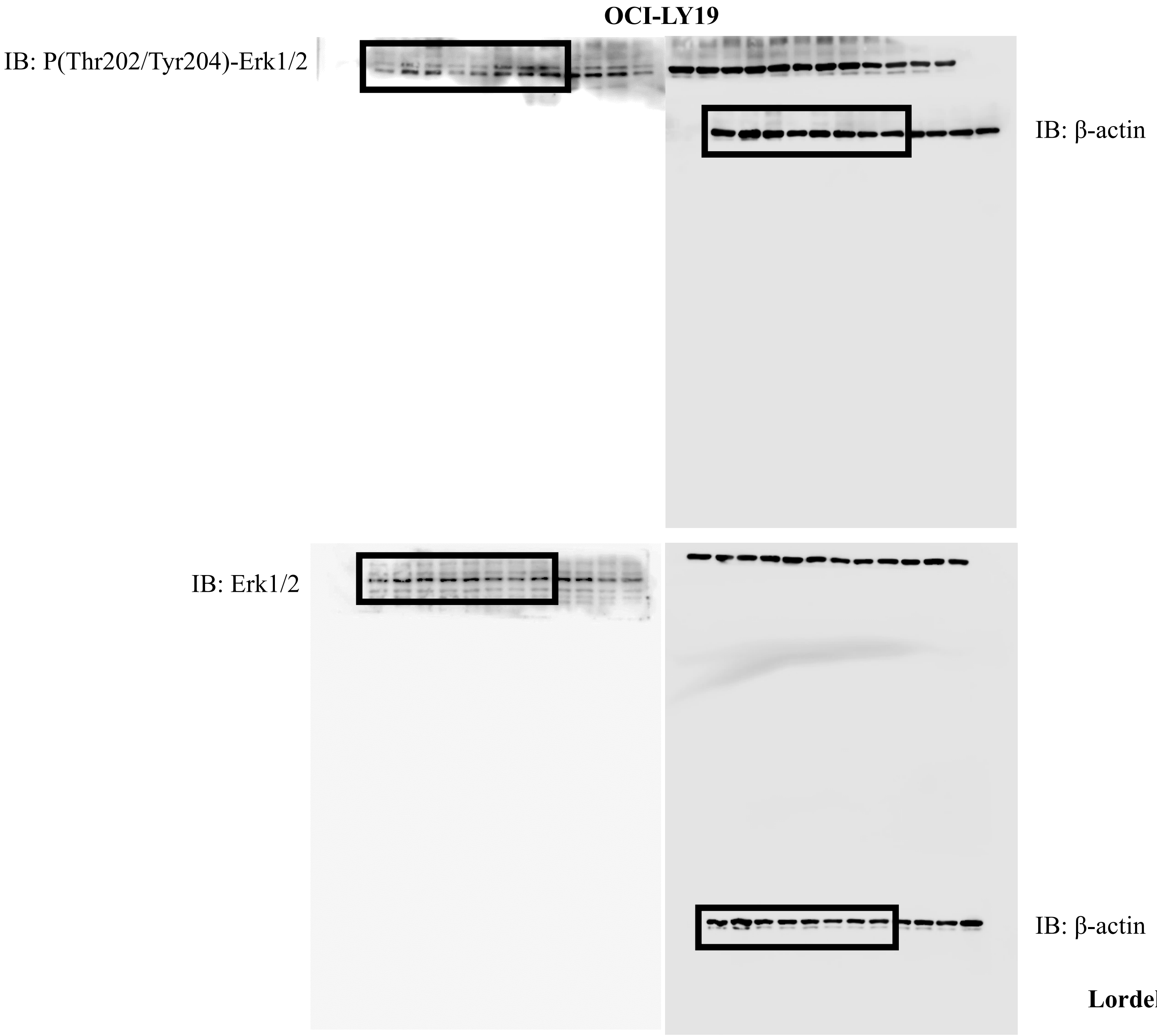

Lordello, L. et al.

Figure 4B

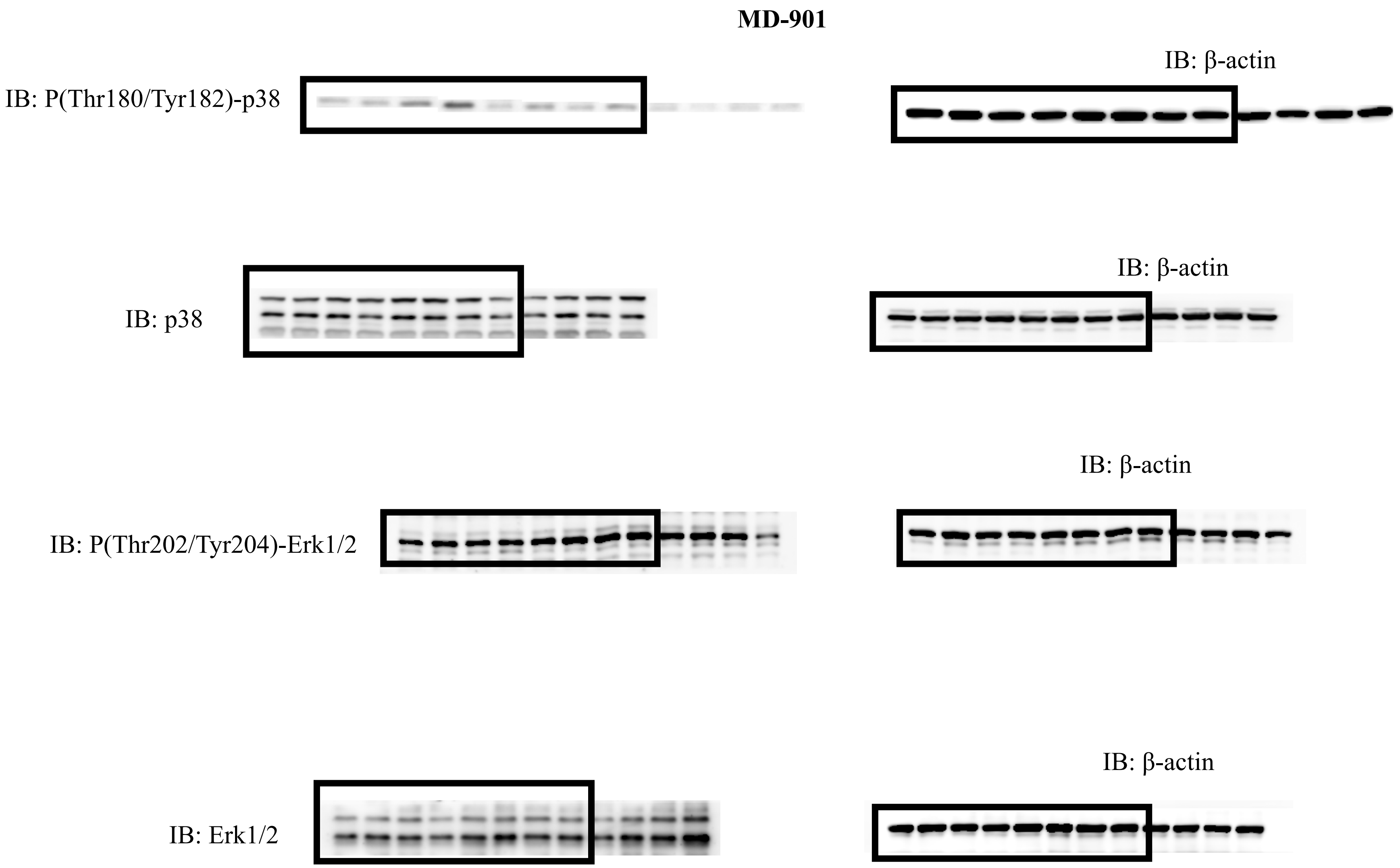

Lordello, L. et al.

Figure 4B

SU-DHL4

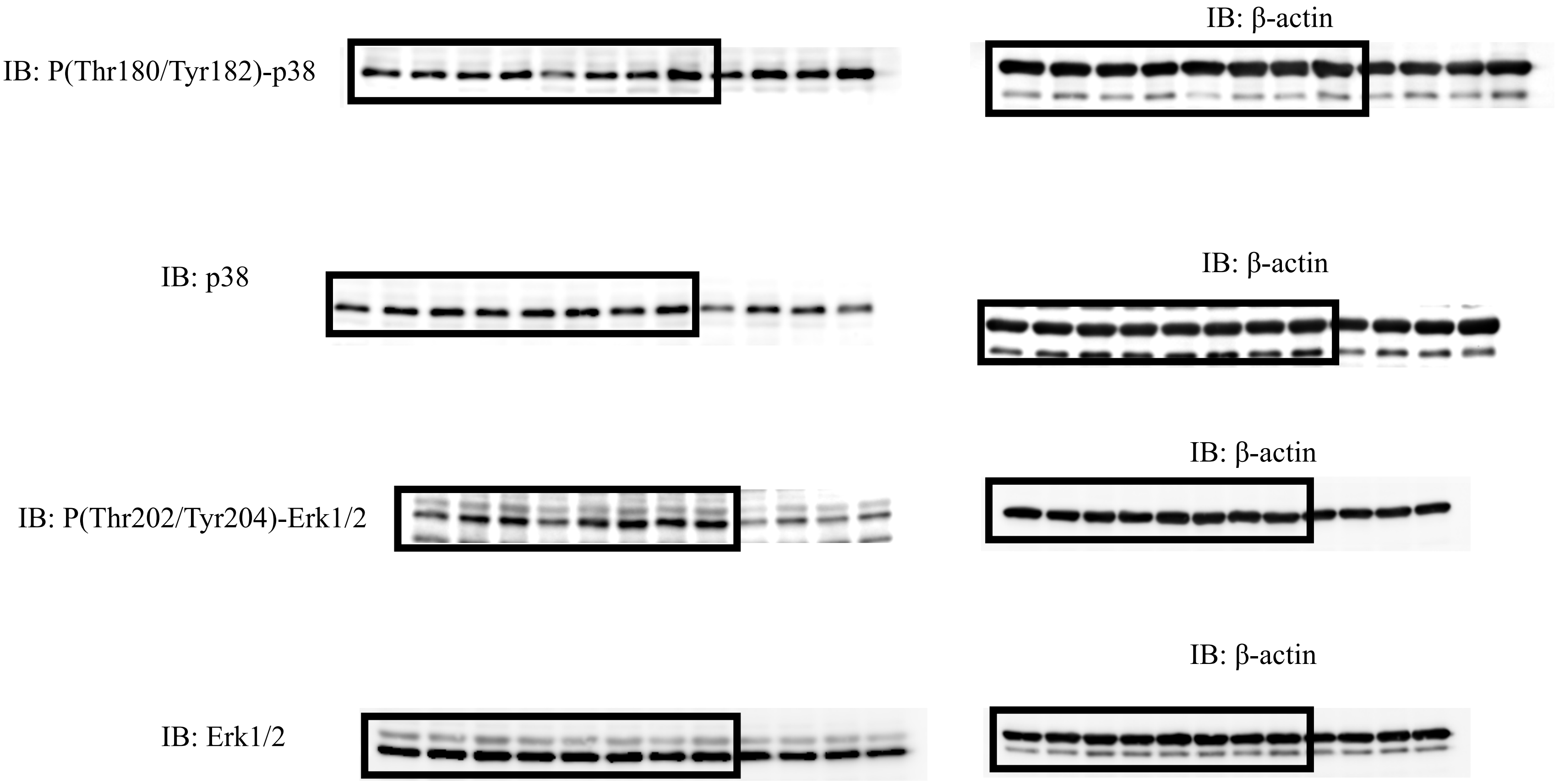

Lordello, L. et al.
